# Screening of Spore-Forming Bacteria with Probiotic Potential in Pristine Algerian Caves

**DOI:** 10.1128/spectrum.00248-22

**Published:** 2022-10-10

**Authors:** Baraa Rehamnia, Natuschka M. Lee, Ramune Kuktaite, Noreddine Kacem Chaouche

**Affiliations:** a Laboratoire de Mycologie, de Biotechnologie et l’Activité Microbienne (LaMyBAM), Université Frères Mentouri Constantine 1, Constantine, Algeria; b Microbial Geoecology and Astrobiology, Department of Ecology and Environmental Science, Umeå University, Umeå, Sweden; c Research infrastructure Fluorescence in situ hybridization (FISH), Chemical Biological Centre, Umeå University, Umeå, Sweden; d Department of Plant Breeding, Swedish University of Agricultural Sciences (Alnarp), Lomma, Sweden; Shenzhen Bay Laboratory

**Keywords:** subsurface, cave geobiology, gliadinase, beta-galactosidase, lactose, probiotics, antibiotic resistance, spore-forming bacteria, *Bacillus*, *Paenibacillus*

## Abstract

The interest and exploration of biodiversity in subsurface ecosystems have increased significantly during the last 2 decades. The aim of this study was to investigate the *in vitro* probiotic properties of spore-forming bacteria isolated from deep caves. Two hundred fifty spore-forming microbes were enriched from sediment samples from 10 different pristine caves in Algeria at different depths. Isolates showing nonpathogenic profiles were screened for their potential to produce digestive enzymes (gliadinase and beta-galactosidase) in solid and liquid media, respectively. Different probiotic potentialities were studied, including (i) growth at 37°C, (ii) survival in simulated gastric juice, (iii) survival in simulated intestinal fluid, and (iv) antibiotic sensitivity and cell surface properties. The results showed that out of 250 isolates, 13 isolates demonstrated nonpathogenic character, probiotic potentialities, and ability to hydrolyze gliadin and lactose in solution. These findings suggest that a selection of cave microbes might serve as a source of interesting candidates for probiotics.

**IMPORTANCE** Previous microbial studies of subsurface ecosystems like caves focused mainly on the natural biodiversity in these systems. So far, only a few studies focused on the biotechnological potential of microbes in these systems, focusing in particular on their antibacterial potential, antibiotic production, and, to some extent, enzymatic potential. This study explores whether subsurface ecosystems can serve as an alternative source for microbes relevant to probiotics. The research focused on the ability of cave microbes to degrade two substrates (lactose and gliadin) that cause common digestive disorders. Since these enzymes may prove to be useful in food processing and in reducing the effect of lactose and gliadin digestion within intolerant patients, isolation of microbes such as in this study may expand the possibilities of developing alternative strategies to deal with these intolerances.

## INTRODUCTION

Subsurface ecosystems are underexplored environments, and they are generally considered extreme, as they are secluded from the surface, nutrient limited, and characterized by lack of light (1, 2). During the last decades, our knowledge about the vast, hidden microbial diversity in the subsurface has expanded considerably. Today, the subsurface is recognized as one of the most diverse habitats for prokaryotes, and current estimates suggest that up to 20% of the total biomass of microbes on Earth is found in the continental subsurface (3). Depending on the characteristics of the subsurface habitat (subseafloor versus terrestrial), different types of functionally active microbial communities can be found (4).

Several studies have shown that caves may serve as excellent models in subsurface research, as caves are easier to access than exhaustive drilling of isolated subsurface areas. Despite the oligotrophic conditions in many caves, the microorganisms in these habitats have been found to be remarkably diverse and exhibit different kinds of adaptations to environmental limitations (5). Thus, microbial survival strategies (e.g., competition and efficient turnover of nutrients) in such environments may present an interesting platform to screen for novel microbes, enzymes, diversified secondary metabolic products, and novel bioactive compounds that may be useful in different types of applications, such as in pharmaceutical and/or food industries (6, 7). Depending on the mineralogical and biogeochemical characteristics of the cave, different kinds of microbes and enzymes have been discovered, such as amylases (8), lipases (9), proteases, cellulases (8), ureases (10), β-glycosidases, phytases, xanthan lyases, and other enzymes involved in carbohydrate catabolism (9). In addition to that, De Mandal et al. (11) studied cave microbial genes encoding enzymes involved in starch degradation (alpha-amylase, glucoamylase, neopullulanase, and pullulanase), cellulose degradation (alpha-glucosidase, endoglucanase, beta-glucanase, beta-glucosidase), hemicellulose degradation (arabinofuranosidase, xylanase, and mannanase), chitin and pectin degradation (chitinase, beta-hexosaminidase, alpha-mannosidase, beta-mannosidase, acetyl-glucosaminidase, and polygalacturonase), and other degradation processes of other categories of carbohydrates (11). These enzymes may be useful in a wide range of biotechnological applications (12), for example, reducing antinutritional factors (e.g., protease and amylase inhibitors in legumes and cereals [13]), as well as in the expanding fields of probiotics in the food industry to provide a beneficial effect in digestive enzymatic diseases (14–16). More specifically, using proteolytic mechanisms of lactic acid bacteria such as serine proteases and several intracellular peptidases might help to hydrolase toxic peptides responsible for gluten-related disorders (17).

Lately, probiotics have received increased interest in the industry, and the demand for novel candidates is therefore constantly increasing (13). Probiotics are living microorganisms that confer health benefits to the host when administered in adequate amounts (18). The majority of commercially exploited probiotic bacteria are non-spore-forming species, such as *Bifidobacterium* spp. and *Lactobacillus* spp. (19). However, spore-forming bacteria may also offer a number of advantages over non-spore-forming bacteria and can therefore serve as an ideal choice for certain products in the global market of probiotics (19). A promising spore-forming genus is *Bacillus*, which consists of nearly 600 known species (https://lpsn.dsmz.de/genus/bacillus), where approximately 1% of these have so far proven to be commercially available for human consumption, namely, B. clausii, B. subtilis, B. coagulans, B. cereus, B. licheniformis, B. polyfermenticus, B. laterosporus, and B. pumilus (20). However, so far, only a few of these studies highlighted the effect of probiotic spore-forming bacteria on gluten intolerance disease (21, 22).

Probiotics used for humans are commonly isolated from human intestinal sources or food, such as fruits and vegetables (23, 24), fermented food (25, 26), fish and seafood (27), and food waste (28). A few studies have also explored natural environments such as soil (29–32). However, to our knowledge, enrichment attempts of probiotic bacteria from subsurface ecosystems like caves and an accompanying study of their health potentialities have not been made so far. Thus, the aim of this study was to explore whether pristine deep subsurface ecosystems can serve as an alternative environmental source for screening bacteria with probiotic potential. Algerian caves were used as an example of a rather unique pristine study site, as these caves have been only scarcely studied and are therefore not heavily contaminated by anthropogenic bacteria. In general, the North African caves are characterized by great geodiversity, vast mineral resources (33), high tectonic activity, and several hot springs. Algeria hosts most of the hypogene caves in Africa (34) and contains many unique, unexplored cave systems on the African continent, such as the two deepest caves, Anou Ifflis (−1,170 m) and Anou Boussouil (−805 m); the largest underground cave network in Africa, Ghar Boumâaza caverns (18,600 m); and one of the deepest gypsum caves in the world, Ghar Kef (2,450 m length and −212 m deep) (35). So far, only one of the Algerian caves (Chaabe Cave) has been microbiologically explored (36). Interestingly, this study explored microorganisms such as actinobacteria that were capable of producing different antibacterial compounds, suggesting that caves can indeed harbor an interesting source of microorganisms with biotechnological potential. To explore whether also bacteria with probiotic potential, such as *Bacillus*, can be found in Algerian caves, we isolated and characterized two hundred 50 strains from sediments in 10 different Algerian caves at different depths, from 0 m to −450 m. Isolated strains were further characterized with regard to different probiotic potentialities, such as antibiotic susceptibility, resistance to gastrointestinal fluid, cell surface characteristics, and ability to degrade gliadin and lactose since these are regarded as beneficial enzymes to combat digestive disorder symptoms (25, 37).

## RESULTS

To screen for the presence of probiotic *Bacillus* strains in subsurface ecosystems, 24 sediment samples were collected from 8 caves in 5 different regions in Algeria (Table 1). Standard physical-chemical analyses showed that 9 of the 10 caves had oligotrophic profiles, as they contained <2 mg of total organic carbon (TOC) per liter. Only one cave (Pirates) was not oligotrophic due to slightly higher contents of organic matter (TOC, 2.72 mg). The pH was neutral in all sediments; however, depending on the cave, the temperature varied from 3.2°C to 25°C, and the humidity varied from 40 to 99.99% (Table 2).

**TABLE 1:** Summary of general characteristics of studied caves, their general profiles, and the selected isolates with probiotic potential

Cave	Code for the cave	GPS coordinates	Region	Depth (m)	Annual precipitation (mm)	Isolate(s) with probiotic potential
Ain Smara 1	AS1	36°15′34.85″N, 06°31′25.78″E	Constantine	−10	630	BS13, BS14, BS16
Ain Smara 2	AS2	36°15′34.85″"N, 06°31′25.78″E	Constantine	−10	630	
Blanche	BL	36°46′12.5″N, 5°04′42.9″E	Bejaia	NA^*a*^	830	
Bouakkous	BOU	35°25′04.4″N, 7°57′49.2″E	Tébessa	−50	830	T1
Boussouil	ANOU	36°28′09.4″N, 4°11′29.5″E	Bouïra	−805	650	J4, SSI5
Ghar Dbaa	GD	36°21′40.8″N, 6°28′29.7″E	Constantine	NA	630	
Ghar Djmaa	GG	36°27′27.2″N, 7°23′08.9″E	Guelma	−129	557	D16, D17
Mchounech 1	MB1	34°57′23.4″N, 6°24′1.6″E	Biskra	NA	141	A8, A9
Mchounech 2	MB2	34°57′41.6″N, 6°00′48.6″E	Biskra	NA	141	D3, F9, F10
Pirate	PR	36°46′19.4″N, 5°05′53.9″E	Bejaia	NA	830	

a NA, not available.

**TABLE 2:** Physicochemical-biological characteristics of the sediments in the 10 different investigated caves

Caves codes	Macrobiodiversity (recorded so far)	Codes for samples	Sampling area depth (m)	Distance from the entrance (m)^*a*^	pH	Organic matter (%)	Total carbonates (%)	Temp (°C)	Humidity (%)
AS1	Spiders	GI, GII	−10	15	8.6 ± 0.02	1.56 ± 0.98	90	13	60.0
AS2	Spiders	SI, SII	−10	20	7.7 ± 0.02	0.83 ± 0.18	100	14	61.0
BL		BL1, BL2	−10	48	9.3 ± 0.00	0.46 ± 0.25	100	13	61.3
BOU		TEB1, TEB2	−50	27	7.1 ± 0.01	0.37 ± 0.31	80	10	72.2
ANOU	Nonidentified insects	BOA1, BOA2	−50		8.0 ± 0.02	0.00	80	7.3	99.99
		BOB1, BOB2	−100		7.15 ± 0.03	0.00	80	4.5	99.99
		BOC1, BOC2	−450		6.91 ± 0.03	0.00	80	3.2	99.99
GD	Bats, moths	GD1, GD2		45	7.5 ± 0.00	2.12 ± 0.31	10	13.5	84.0
GG	Moths	CAS2, CAS1		68	8.3 ± 0.00	0.18 ± 0.12	0	14.1	73.3
MB1	Reptiles	BM1, BM1		9	8.1 ± 0.00	0.09 ± 0.06	40	23	43.2
MB2	Reptiles	BMS1, BMS2		11	7.4 ± 0.12	0.00	60	25	42.2
PR	Bats	PR1, PR2		39	8.0 ± 0.00	4.69 ± 0.18	40	19	63.3

a All of the samples were taken from the dark zone.

### Isolation and screening.

**FIG 1:**
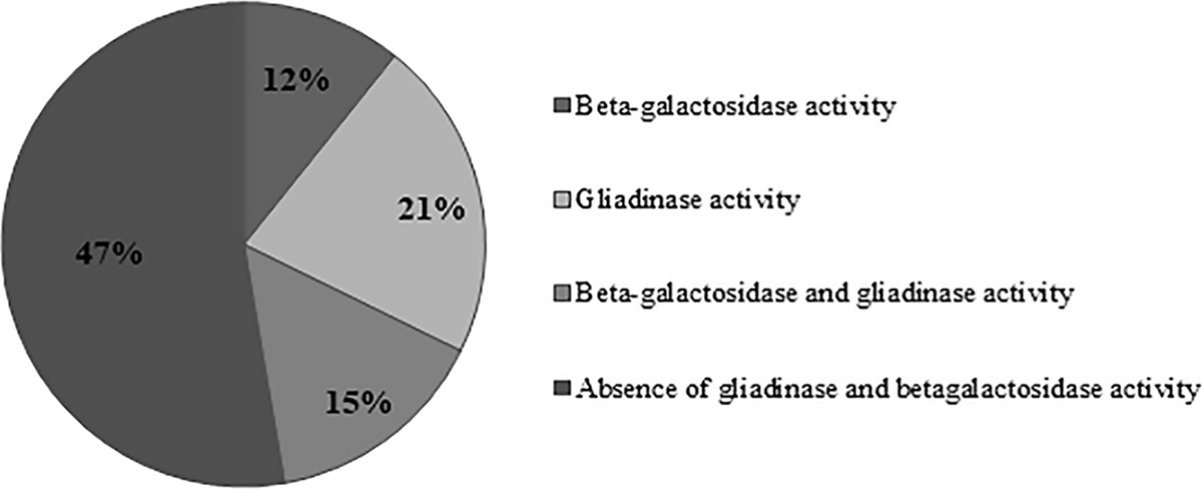
Percentages of enzymatic screening results for 250 isolates.

**FIG 2:**
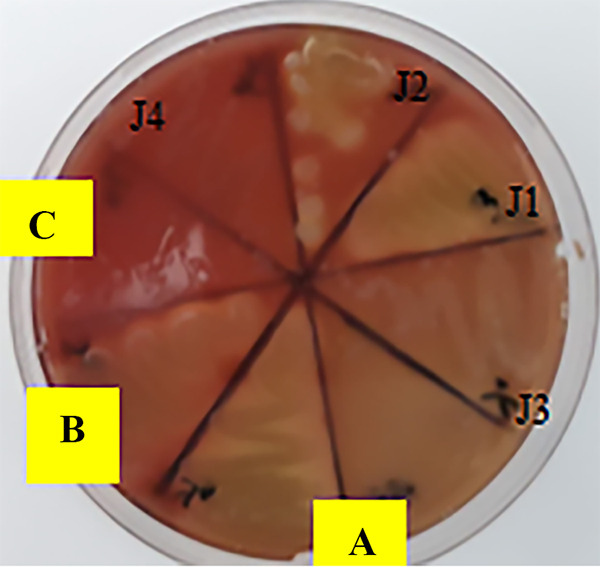
Examples of examined hemolysis patterns for the isolates J1, J2, J3, and J4, sorted by observing hydrolysis zones, alpha-hemolysis (greenish color, isolate Y9), beta-hemolysis (clear zone, isolate Y7), and gamma-hemolysis (no reaction, isolate Y8).

**FIG 3:**
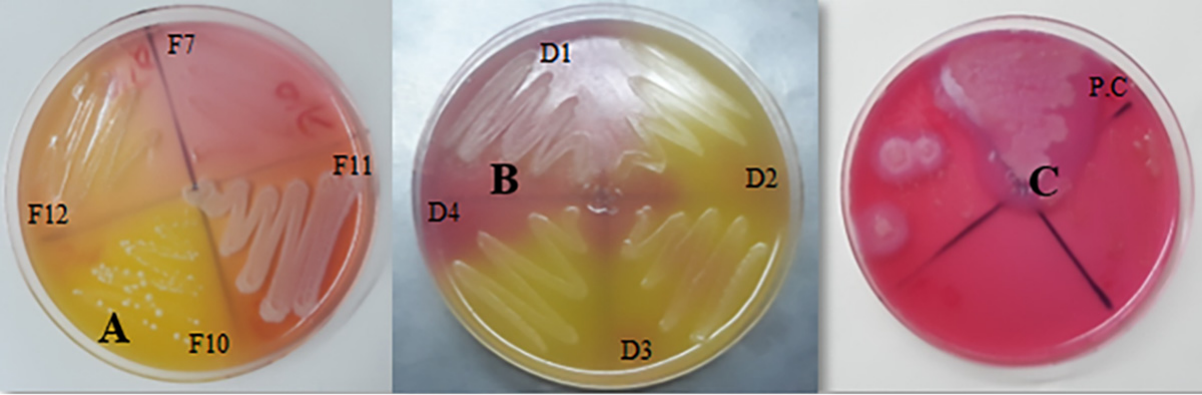
Example of lecithinase test on 9 of the isolates (F7, F10, F11, F12, D1, D2, D3, D4, and positive control [P.C.]). (A) Yellow, no reaction (negative lecithinase) (isolate F10); (B) pink with a halo surrounding colonies (positive lecithinase) (isolate D1); (C) positive-control B. cereus.

**FIG 4:**
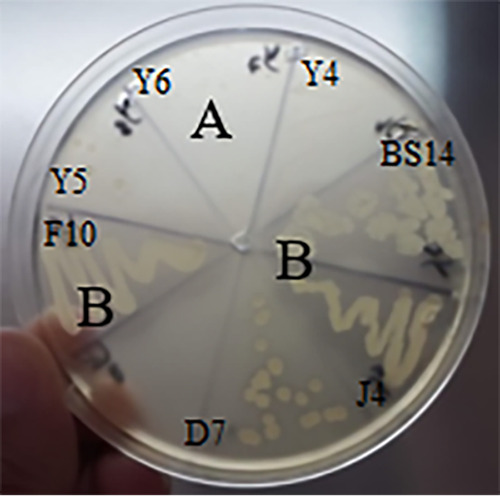
Example of gliadinase screening test on 7 isolates (BS14, D7, F10, J4, Y4, Y5, and Y6). (A) Absence of gliadin degradation (isolate Y5). (B) Clear halos surrounding colonies (strains BS14, D7, J4, and F10) show gliadin degradation.

**FIG 5:**
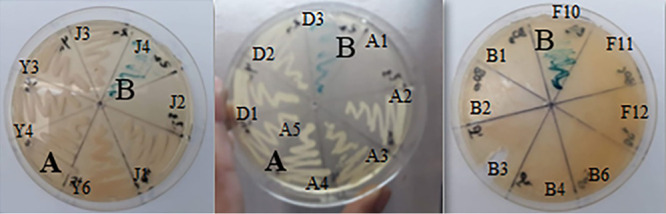
Example of beta-galactosidase test on 23 isolates (J1, J2, J3, J4, Y3, Y4, Y6, A1, A2, A3, A4, A5, D1, D2, D3, B1, B2, B3, B4, B6, F10, F11, and F12). (A) No beta-galactosidase production; (B) colonies in blue, production of beta-galactosidase (strains J4, D3, and F10).

After heating treatment, 250 bacterial isolates were obtained out of 24 cave sediment samples (BM1, BM2, BMS1, BMS2, BOA1, BOA2, BOB21, BOB2, BOC1, BOC2, CAS2, CAS1, GD1, GD2, GI, GII, TEB1, TEB2, PR1, PR2, SI, SII, BL1, and BL2) (Table 2). All isolates were tested for their probiotic potential, employing different test methods as described in the following sections. All selected strains showed proteolytic activity (Fig. 1). Seventy-four isolates showed gamma-hemolysis patterns and negative lecithinase reactions (Fig. 2 and 3). Thirty-four isolates showed significant gliadinase activity based on the formation of an opaque color around their colonies (Fig. 4). Twenty-four isolates were regarded preliminarily as isolates with beta-galactosidase activity due to the formation of blue colonies (which could reflect the presence of either extra- or intracellular beta-galactosidase) (Fig. 5). Based on these different prescreening tests, in particular, their ability to hydrolyze gliadin and lactose, 13 strains were selected from these 24 isolates for further evaluation of their probiotic potential. All details of the results are presented in reference 38.

### Beta-galactosidase assay.

**FIG 6:**
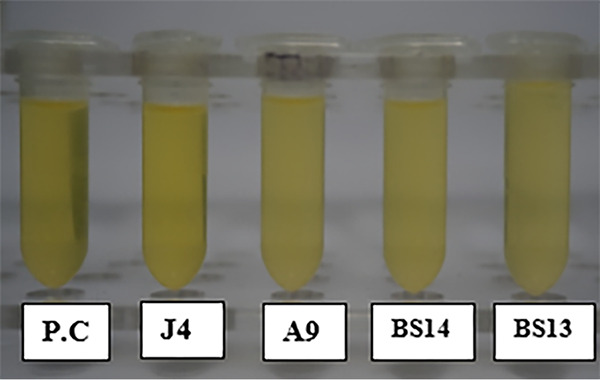
Example of positive results for the beta-galactosidase assay (for the isolates J4, A9, BS14, and BS13). P.C., positive control (E. coli ATCC 25922).

Four strains (BS13, BS14, J4, and A9) out of the 13 tested showed considerable ortho-nitrophenyl-*p*-d-galactopyranoside (ONPG) activity (Fig. 6). Strain BS13 produced the largest amount of beta-galactosidase (429.5 Miller units [MU]), while the other tested strains produced somewhat lower values: BS14 produced 317.81 MU, J4 produced 224.5 MU, and A9 produced 181.25 MU. The rest of the tested strains showed either none or considerably lower concentrations of beta-galactosidase (Table 3).

**TABLE 3:** Summary information about the 13 selected strains^*a*^

DSM no.	Strain	Cave	β-Hemolysis	Lecithinase	Beta-galactosidase screening	Gliadinase screening	Beta-galactosidase assay (MU)	Gliadinase assay	Gliadinase production in gel	Antibiotic susceptibility (dose [μg])	Survival in low pH (%)	Survival in simulated gastric juice (%)	Survival in bile salts (%)	Survival in intestinal fluid (%)	Auto-aggregation (%)	Coaggregation (%)	Adhesion to xylene (%)	Adhesion to chloroform (%)	GenBank accession no.
10172	A8	Mchounech 1	−	−	+	+	−	+	+	Ampicillin (2), ciprofloxacin (1), erythromycin (10), vancomycin (5), imipenem (10)	100	100	98.59	100	96.62	76.79	5.13	1.06	OL823170
19772	A9	Mchounech1	−	−	+	+	181.25	+	+	Amikacin (30), ciprofloxacin (1), erythromycin (10), rifampin (15), vancomycin (5), imipenem (10)	100.14	100	100	100	100.00	92.26	1.16	1.16	OL823171
10764	F9	Mchounech 2	−	−	+	+	−	+	+	Ampicillin (2), ciprofloxacin (1), erythromycin (10), gentamicin (10), vancomycin (5), imipenem (10)	0.3		99.07	99.67	100.00	73.21	10.76	23.08	OL823176
10765	F10	Mchounech 2	−	−	+	+	−	+	+	Ciprofloxacin (1), imipenem (10)	99.99	100	99.9	100	100.00	79.30	100.00	100.00	OL823177
10771	D3	Mchounech 2	−	−	+	+	−	+	+	Ampicillin (2), ciprofloxacin (1), erythromycin (10), vancomycin (5), imipenem (10)	100.17	100	100	100	100.00	84.21	100.00	100.00	OL823173
10769	Dj16	Ghar Djmaa	−	−	+	+	−	+	+	Erythromycin (10), imipenem (10)	23.65		100	98.96	100.00	90.13	1.01	26.26	OL823174
10770	Dj17	Ghar Djmaa	−	−	+	+	−	+	+	Ampicillin (2), amoxycillin (10), ciprofloxacin (1), erythromycin (10), rifampin (15), imipenem (10)	99.99	100	83.51	100	91.84	85.86	1.25	15.00	OL823175
10774	BS13	Ain Smara 1	−	−	+	+	429.5	+	+	Amikacin (30), ciprofloxacin (1), vancomycin (5), imipenem (10)	97.14	100	94.27	100	100.00	88.30	100.00	100.00	OM146734
10775	BS14	Ain Smara 1	−	−	+	+	317.81	+	+	Amikacin (30), ciprofloxacin (1), vancomycin (5), imipenem (10)	100	100	100	98.83	100.00	82.84	100.00	100.00	OM250505
10773	BS16	Ain Smara 1	−	−	+	+	−	+	+	Ciprofloxacin (1), imipenem (10)	100.09	99.99	100	100	100.00	79.61	7.95	14.77	OL823172
10766	J4	Boussouil	−	−	+	+	224.5	+	+	Ampicillin (2), amoxycillin (10), ciprofloxacin (1), rifampin (15), imipenem (10)	62.96	100	100	100	96.60	87.38	40.90	100.00	OL823178
19768	T1	Bouakkous	−	−	+	+	−	+	+	Ciprofloxacin (1), rifampin (15), vancomycin (5), imipenem (10)	100.08	100	100	100	93.03	73.25	7.95	14.77	OL823180
19767	SSI5	Boussouil	−	−	+	+	−	+	+	Ciprofloxacin (1), gentamicin (10), rifampin (15), imipenem (10)	40.9	100	100	100	100.00	82.55	40.91	99.60	OL823179

a +, enzyme found; −, enzyme not found.

### Gliadin degradation in solution.

**FIG 7:**
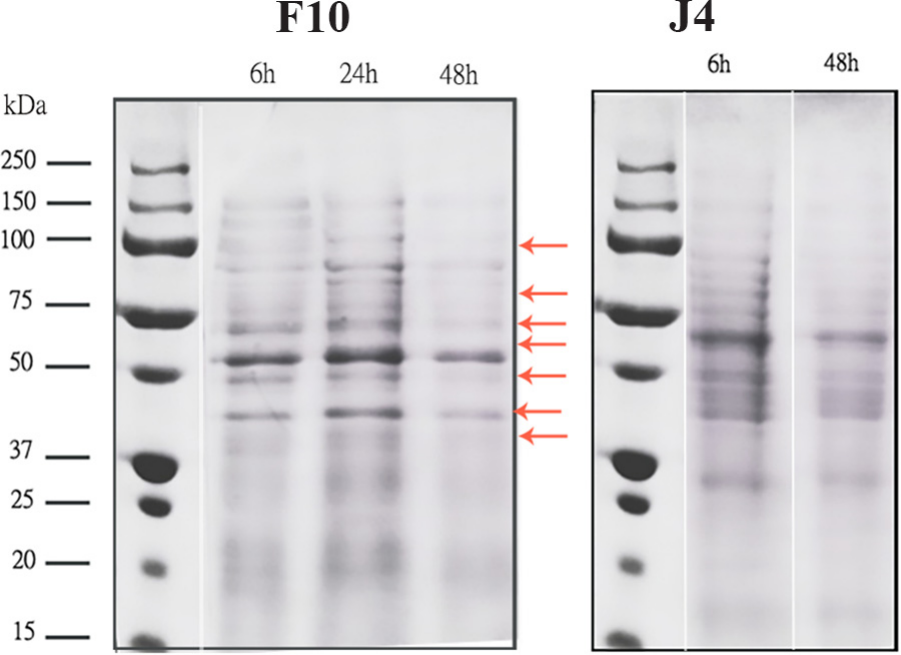
Example of SDS-PAGE profiles of gliadin degradation for the strains F10 and J4. Gliadin was used as the sole nitrogen source. The intensity of bands ranging from ~30 to ~50 (representing α-, β-, and γ-gliadins) decreased markedly, while a lower pattern of degradation in bands between ~50 and 100 (related to the ω-gliadins) was noticed. A strain (Y5) that did not show gliadin degradation ability in gliadin plates served as negative control.

All 13 strains exhibited different levels of gliadin degradation, with similar patterns in two different media, with a substantial degradation of ω-gliadins (between ~50 and 100 kDa) and a noticeable degradation of α-, β-, and γ-gliadins (ranging from ~30 to 50 kDa) (Fig. 7).

### Degradation of gliadin in gel.

**FIG 8:**
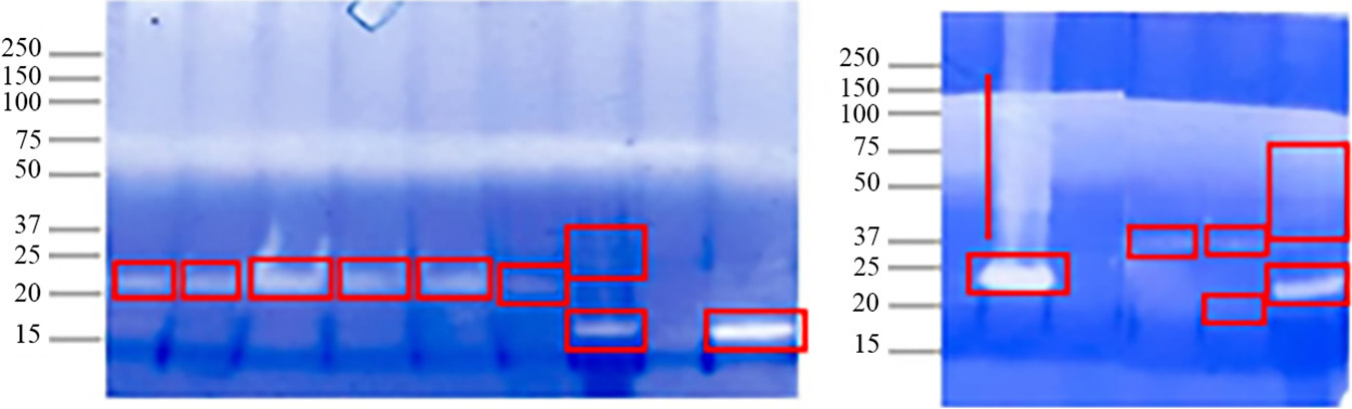
Gliadin-based zymogram for the isolates (A8, A9, F9, F10, Dj16, Dj17, D3, BS13, BS14, BS16, J4, SSI5, T1, and Y5). Selected bacterial solutions were subjected to zymography. Hydrolysis was noted by the appearance of clear bands after Coomassie staining since the gel contained gliadin as a substrate. Vertical lines demonstrate clearing areas where no individual bands can be clearly identified.

For all 13 strains, in-gel zymography revealed clearing bands with different intensities showing gliadin-degrading enzymes (gliadinases) between the ~15-kDa to ~50-kDa regions (a broad molecular weight range). (Fig. 8). According to Alvarez-Sieiro et al. (39), clearing areas indicate either a strong hydrolytic activity or the existence of various activities of proteins with similar molecular weights.

### *In vitro* survival in simulated gastrointestinal fluids.

Nine (A8, A9, F10, D3, BS13, BS14, BS16, J4, and SSI5) out of 13 tested strains showed a high survival rate under simulated gastric conditions (99 to 100%). Strain Dj16 revealed viability count with a significant difference (*P = *0.05) variability, showing the highest reductions in cell viability, with low pH and no resistance to simulated gastric juice (Table 4). All strains, except A9, showed different resistance patterns to bile salts and simulated intestinal fluid, with a survival rate ranging between 83.51% and 100% (Table 5).

**TABLE 4:** *In vitro* acid tolerance and survival after exposure to simulated gastric juice for 13 selected strains^*a*^

Strain	Viable counts at pH 1.5 (log CFU/mL) at:		pH tolerance (%)	Viable counts with gastric conditions (log CFU/mL) at:		Survival rate (%)
	0 h	3 h		0 h	3 h	
A8	7.66 ± 0.01	7.67 ± 0.01	100.00	7.66 ± 0.01	7.66 ± 0.01	100.00
A9	7.06 ± 0.01	7.05 ± 0.00	100.14	7.06 ± 0.01	7.06 ± 0.01	100.00
F9	7.57 ± 0.01^*b*^	5.03 ± 0.02^*b*^	0.30	7.57 ± 0.01^*b*^	—^*b*^	—^*b*^
F10	7.71 ± 0.01	7.71 ± 0.00	99.99	7.71 ± 0.01	7.71 ± 0.01	100.00
D3	6.79 ± 0.03	6.81 ± 0.02	100.17	6.79 ± 0.03	6.79 ± 0.03	100.00
Dj16	6.69 ± 0.02^*b*^	6.04 ± 0.05^*b*^	23.65	6.69 ± 0.02^*b*^	—^*b*^	—^*b*^
Dj17	6.56 ± 0.05	6.55 ± 0.05	99.99	6.56 ± 0.05	5.88 ± 0.43	100.00
BS13	6.99 ± 0.00	6.98 ± 0.01	97.14	6.99 ± 0.00	6.98 ± 0.01	100.00
BS14	6.64 ± 0.04	6.64 ± 0.05	100.00	6.64 ± 0.04	6.64 ± 0.04	100.00
BS16	7.01 ± 0.03	7.03 ± 0.03	100.09	7.01 ± 0.03	7.01 ± 0.03	99.99
J4	7.12 ± 0.04^*b*^	6.92 ± 0.00^*b*^	62.96	7.12 ± 0.04	6.91 ± 0.01	100.00
T1	7.71 ± 0.00	7.71 ± 0.00	100.08	7.71 ± 0.00	7.71 ± 0.00	100.00
SSI5	6.57 ± 0.07^*b*^	6.19 ± 0.13^*b*^	40.90	6.57 ± 0.07	6.55 ± 0.02	100.00

a Acid tolerance (%) = (CFU with bile/CFU without bile) × 100. Survival rate of 100% means the survival rate is not affected. All results are expressed as mean ± standard deviation (*n* = 3). —, no tolerance.

b Significant differences (*P* < 0.05).

**TABLE 5:** *In vitro* tolerance and survival rates after exposure to simulated intestinal fluid for 13 selected strains^*a*^

Strain	Viable counts in 1% ox gall (log CFU/mL) at:		Bile tolerance	Viable counts with intestinal conditions (log CFU/mL) at:		Survival rate (%)
	0 h	4 h		0 h	4 h	
A8	8.12 ± 0.00	8.00 ± 0.00	98.59	8.12 ± 0.00	7.81 ± 0.20	100.00
A9	7.35 ± 0.01	7.63 ± 0.22	100.00	7.35 ± 0.01	7.34 ± 0.01	100.00
F9	8.29 ± 0.03	8.21 ± 0.04	99.07	8.29 ± 0.03	8.32 ± 0.01	99.67
F10	8.27 ± 0.01	8.26 ± 0.00	99.90	8.27 ± 0.01	8.17 ± 0.07	100.00
D3	7.36 ± 0.16	7.73 ± 0.21	100.00	7.36 ± 0.16	7.30 ± 0.13	100.00
Dj16	6.00 ± 0.03	6.65 ± 0.24	100.00	6.00 ± 0.03	6.06 ± 0.11	98.96
Dj17	8.27 ± 0.01^*b*^	6.91 ± 0.58^*b*^	83.51	8.27 ± 0.01	7.80 ± 0.42	100.00
BS13	8.26 ± 0.00	7.79 ± 0.20	94.27	8.26 ± 0.00	8.14 ± 0.16	100.00
BS14	7.58 ± 0.13	8.03 ± 0.48	100.00	7.58 ± 0.13	7.67 ± 0.01	98.83
BS16	7.68 ± 0.04	8.28 ± 0.05	100.00	7.68 ± 0.04	7.64 ± 0.01	100.00
J4	7.79 ± 0.01	7.86 ± 0.06	100.00	7.79 ± 0.01	7.79 ± 0.00	100.00
T1	8.20 ± 0.01	8.40 ± 0.25	100.00	8.20 ± 0.01	8.11 ± 0.11	100.00
SSI5	7.78 ± 0.03	8.09 ± 0.42	100.00	7.78 ± 0.03	7.70 ± 0.17	100.00

a Bile tolerance (%) = (CFU with bile/CFU without bile) × 100. Survival rate of 100% means the survival rate is not affected. All results are expressed as mean ± standard deviation (*n* = 3).

b Significant differences (*P* < 0.05).

### Antibiotic susceptibility.

The antibiotic susceptibility to 11 different antibiotics of the 13 selected strains was assessed according to European Food Safety Authority (EFSA) recommendations (40). All selected strains, except strain Dj16, showed sensitivity to imipenem (10 μg) and ciprofloxacin (1 μg). Six strains (F10, Dj16, Dj17, BS16, SSI5, and J4) were resistant to vancomycin (5 μg) (Table 6).

**TABLE 6:** Antibiotic susceptibility of the 13 selected strains^*a*^

Antibiotic (dose [μg])	Susceptibility of strain:												
	A8	A9	D3	F9	F10	BS13	BS14	DJ16	DJ17	BS16	T1	SSI5	J4
Amikacin (30)	R	S	R	S	R	S	S	R	R	R	R	R	R
Ampicillin (2)	S	R	S	R	R	R	R	R	S	R	R	R	S
Amoxycillin (10)	R	R	R	R	R	R	R	R	S	R	R	R	S
Cefazolin (30)	R	R	R	R	R	R	R	R	R	R	R	R	R
Ciprofloxacin (1)	S	S	S	S	S	S	S	R	S	S	S	S	S
Erythromycin (10)	S	S	S	S	R	R	R	S	S	R	R	R	R
Gentamicin (10)	R	R	R	S	R	R	R	R	R	R	R	S	R
Oxacillin (5)	R	R	R	R	R	R	R	R	R	R	R	R	R
Rifampin (15)	R	S	R	R	R	R	R	R	S	R	S	S	S
Vancomycin (5)	S	S	S	S	R	S	S	R	R	R	S	R	R
Imipenem (10)	S	S	S	S	S	S	S	S	S	S	S	S	S

a Antibiotic susceptibility of selected bacteria results were expressed as follows: S, susceptible, and R, resistant. Diameters were measured according to EFSA breakpoints (40).

### Auto-aggregation and coaggregation.

**FIG 9:**
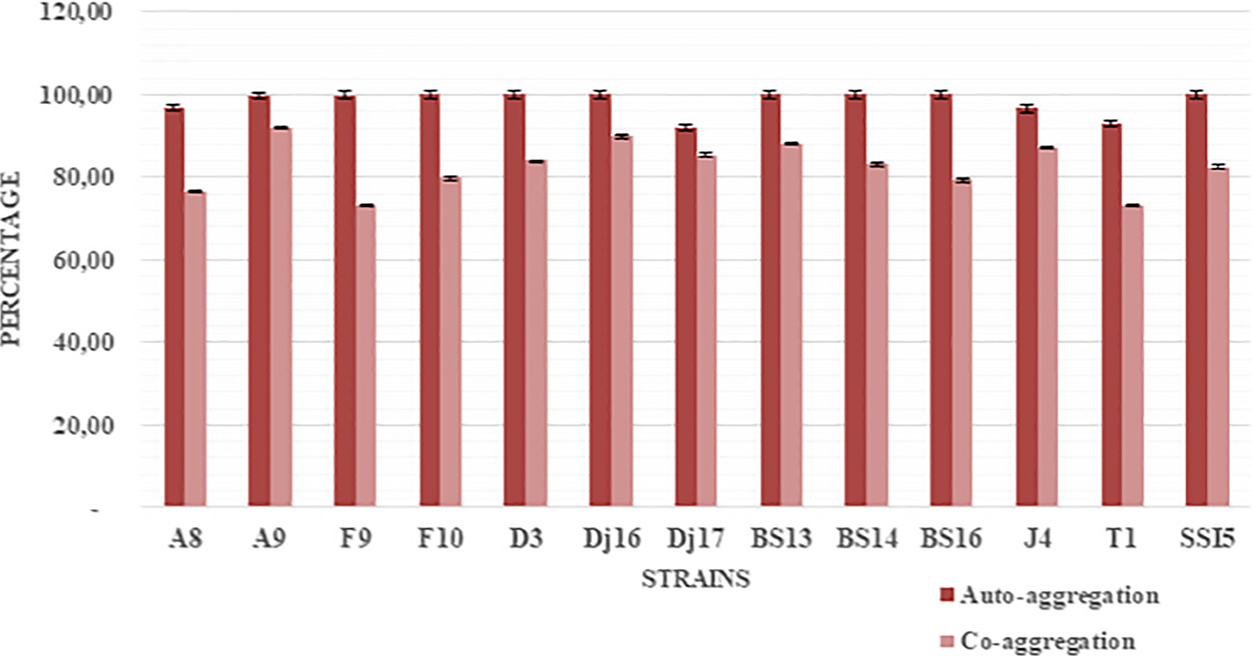
Auto-aggregation and coaggregation of the 13 selected strains.

Auto-aggregation and coaggregation were studied as an index of cell wall properties. The highest values of coaggregation with Escherichia coli were observed for 8 of the 13 strains (A9, F9, D3, Dj16, BS13, BS14, BS16, and SSI5), with a percentage of 100%. The remaining strains showed lower percentages of auto-aggregation, ranging between 72% and 92% (Fig. 9).

### Cell surface hydrophobicity.

**FIG 10:**
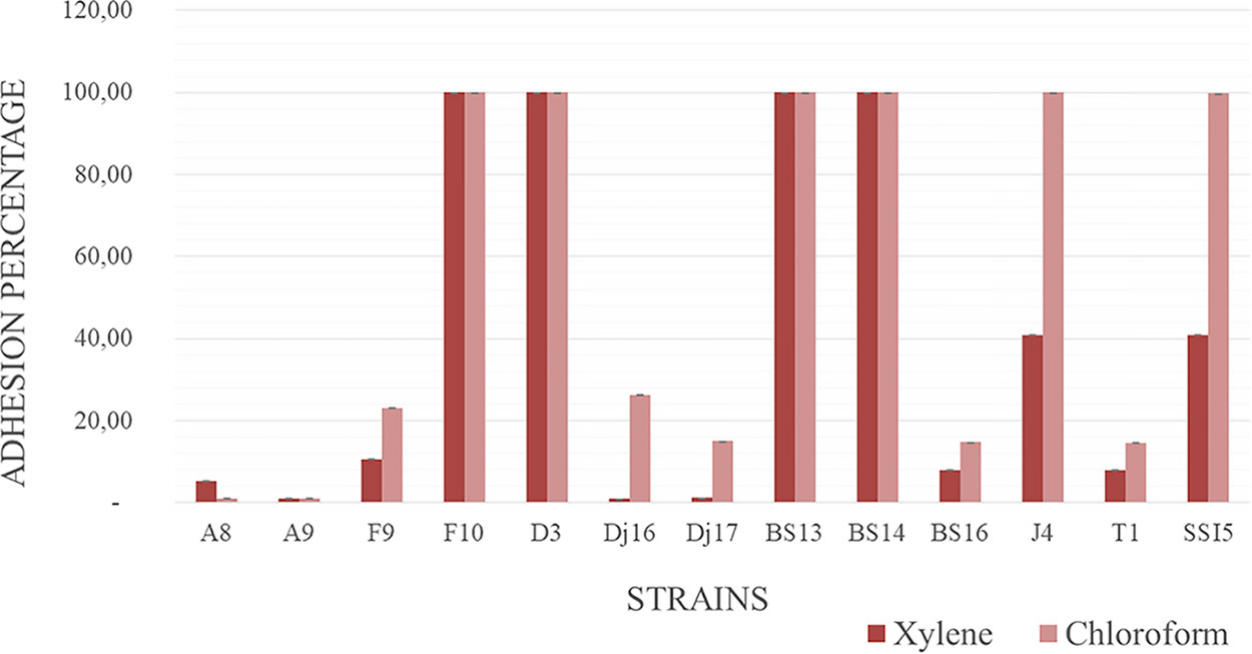
Hydrophobicity and electron receptors of the 13 selected strains.

Four strains (F10, D3, BS13, and BS14) out of the 13 tested strains were hydrophobic, exhibiting an adhesion percentage of 100% to xylene and chloroform. All four strains showed a high affinity for chloroform (strong electron acceptors) and a significant affinity for xylene. Seven of the strains (A8, A9, F9, Dj16, Dj17, BD16, and T1) showed a hydrophilic character, with < 50% adhesion to xylene and chloroform, respectively (Fig. 10).

### *In situ* identification of strain purity and taxonomic affiliation via FISH.

All 13 strains showed weak fluorescence *in situ* hybridization (FISH) signals when pretreated only with lysozyme prior to the hybridization procedure, as suggested in reference 41. However, after employing a higher concentration of lysozyme, including an additional enzyme (achromopeptidase), and expanding the incubation time to 30 min at 37°C, prior to hybridization, stronger FISH signals were obtained with the gene probes EUB338 and LGC354B. The controls suggested that the natural background autofluorescence is negligible and that the fluorochrome used for the gene probes does not cause unspecific reactions with the background matrix (results not shown).

### Phenotypic characteristics.

All 13 strains showed positive Gram-characteristics based on the Gram stain and the KOH test, produced spores, and had a typical rod-shaped morphology with an average diameter of 0.5 to 1.0 μm and length of 1.0 to 4.0 μm (results not shown). All of the following 13 strains were isolated from sediments in oligotrophic caves: A8 and A9 from Mchounech 1; BS13, BS14, and BS16 from Ain Smara 1; Dj16 and Dj17 from Ghar Djemaa; F9, F10, and D3 from Mchounech 2; J4 and SSI5 from Boussouil; and T1 from Bouakkous.

### Phylogenetic analysis.

**FIG 11:**
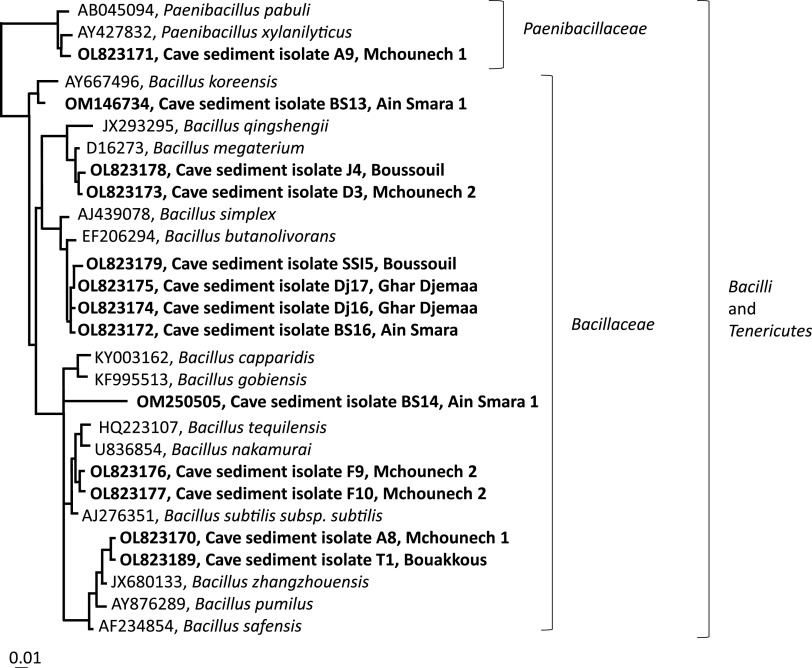
Phylogenetic tree (unrooted) showing the tentative 16S rRNA gene relationships between the 13 isolates retrieved in this study and their closest relationships to 15 other sequences from culturable organisms within the LTP database, version 132 (https://www.arb-silva.de/no_cache/download/archive/previous_living_tree/).

The partial 16S rRNA gene sequences (between 300 and 1,000 nucleotides long) of all 13 isolates were affiliated with different taxa in *Bacilli* within *Firmicutes* (Fig. 11). Phylogenetic evaluations with regard to near-full (>1,200 nucleotides), high-quality 16S rRNA gene sequences of culturable organisms from the SILVA database revealed that the 13 species formed 2 distinctive groups (Fig. 11).

The first is the *Paenibacillus* group, encompassing only one isolate, A9 (from the cave Mchounech), showing the highest similarity to the culturable species Paenibacillus pabuli (98.5%) in the LTP database (version 132), and Paenibacillus taichungensis (98.6%) in the *Bacillales* database (SILVA SSU Parc database [version 138.1]).

The second is the *Bacillales* group, distributed into three subgroups as follows. Subgroup 1 encompasses only one isolate, BS13 (from the cave Ain Smara 1), showing the highest similarity (100%) to the culturable species Bacillales capparidis and Bacillales gobiensis in the LTP database (version 132) and to Bacillales indicus (>99%) in the *Bacillales* database (SILVA SSU Parc database [version 138.1]). Subgroup 2 encompasses four of the isolates, retrieved from two different caves, distributed into two further groups. F9 and F10, retrieved from the same cave (Mchounech), showed the highest similarities to B. subtilis in the LTP database (version 132) and to Bacillus tequilensis in the SILVA SSU Parc databases (>99%, respectively). T1 and A8, representing two different caves (Bouakkous and Mchounech, respectively), showed highest similarities to Bacillus zhangzhouensis and Bacillus pumilus (>98%, respectively). Subgroup 3, encompassing six of the isolates, retrieved from three different caves, was distributed in two further groups, D3 and J4, representing two different caves (Mchounech and Boussouil, respectively), and SSI5, Dj16, Dj17, and BS16, representing two different caves (Boussouil and Ghar Djemaa). All showed significant similarities to Bacillus megaterium, Bacillus simplex, and some other *Bacillus* strains (from 94% to 99% similarity). Interestingly, 3 of the unculturable organisms closely related to the 13 isolates in this study had been retrieved from the subsurface or other cave/mine environments, and the gene sequences from other related organisms had been retrieved from various environments in different terrestrial and aquatic ecosystems.

## DISCUSSION

The purpose of this study was to explore the potential presence of bacteria with probiotic potential in pristine subsurface ecosystems. Different safety screening tests were performed on 250 isolates, and 13 strains were chosen for further study based on their ability to produce beta-galactosidase and gliadinase and criteria stated by the Qualified Presumption of Safety (QPS) assessment framework (42) and the Joint FAO/WHO Expert Consultation (18). Based on partial 16S rRNA gene analyses for brief tentative phylogenetic analyses, all 13 strains belonged to *Bacilli* within *Firmicutes*, showing the highest similarities to different *Bacillus* strains (e.g., B. simplex, B. megaterium, B. safensis, B. pumilus, B. zhangzhouensis, B. tequilensis, B. koreensis, and B. indicus) and one *Paenibacillus* strain (P. xylanilyticus). According to reference 43, *Bacillus* species are ubiquitous in nature, including in caves, such as in speleothems (44), sediments (45), and air (46). Several of these cave *Bacillus* species are involved in biomineralization (44) and bioprecipitation (47), such us B. simplex, B. gaemokensis, B. subtilis, B. thuringiensis, B. albus, B. cereus, B. anthracis, B. weihenstephanensis (44, 48), B. megaterium (49), and other *Bacillus* strains yet to be identified at the species level (50). New *Bacillus* species have also been found in caves, such as B. cavernae (45), B. antri (51), and B. onubensis sp. nov. (46). Thus, this study tentatively indicates that at least some additional *Bacillus*- and *Paenibacillus*-affiliated strains can be found in Algerian cave systems.

In our study, only BS14, BS13, J4, and A9 produced considerable amounts of beta-galactosidase; the remaining strains showed weak or no beta-galactosidase activity based on the conditions tested. However, it cannot be excluded that these strains may also produce larger amounts of beta-galactosidase if the incubation conditions are modified (e.g., by optimizing carbon source or other parameters) (37).

Interestingly, all selected strains were able to hydrolyze ~2 g/L gliadin within 24 h. They also showed a wide spectrum of peptidase activities, and they can degrade raw and acid-treated gliadin of various molecular weights between 30 and 50 kDa, which is of particular interest since the molecular weight of the most immunogenic gluten subunits (α-gliadin epitopes) is approximately 37 kDa (52). These results were similar to those reported in reference 39, where B. subtilis and Bacillus amyloliquefaciens showed the ability to degrade gliadin and use it as a sole nitrogen source. This is further supported by recent studies demonstrating that several different *Bacillus* species can produce extracellular gliadinases (15, 39, 53).

All 13 selected strains demonstrated significant gliadinase activity by use of in-gel zymography, with different intensities ranging from approximately 15 to 150 kDa. This may suggest the presence of multiple peptidases capable of gliadin hydrolysis. However, since in-gel zymography can be inaccurate in the determination of the molecular size of some peptidases due to decreased mobility of proteins in acrylamide gel copolymerized with protein substrate (54), this technique was, in this study, merely used as an additional confirmation of the actual presence of gliadin hydrolytic enzymes. The selected strains were also able to resist acidic gastrointestinal tract conditions, which may indicate that cave microorganisms are remarkably diverse and exhibit high adaptation behaviors to environmental limitations (55).

Antibiotic resistance is a common ancient, widespread phenomenon in environmental bacteria, deeply implanted in the microbial pangenome (56). Several reports showed that nonpathogenic environmental bacteria foster various antibiotic resistance mechanisms and genes (57, 58) and that antibiotic resistance can be detected also in microorganisms isolated from extreme environments absent of human influence, such as the deep ocean (59) and the subsurface (56, 60). In the present study, all isolates showed resistance to penicillin antibiotics (30 μg cefazoline and 5 μg oxacillin), which is a common trait in *Bacillus* species because of the presence of β-lactamase genes (61). The latter may cause enzymatic degradation of antibiotics or modification of penicillin binding proteins (PBPs) (62). In addition to this, antibiotic resistance is rather common in Gram-positive bacteria due to gene acquisition (through plasmids, foreign DNA recombination, or mutations by changes in the native PBP genes) (63). Hence, since probiotics are usually recommended alongside antibiotic therapy to circumvent gastrointestinal distress, such as antibiotic-induced diarrhea, some resistance to commonly administered antibiotics is desirable (64). However, it is nevertheless preferable that probiotics harbor few antibiotic resistance genes to avoid transferring them to other intestinal microflora, including pathogens (65, 66).

Interestingly, the 13 selected strains demonstrated sensitivity to the broad-spectrum antibiotic imipenem (10 μg), and 10 of the strains demonstrated sensitivity to the broad-spectrum antibiotic ciprofloxacin (1 μg). Six of the strains demonstrated sensitivity resistance to vancomycin (5 μg) employed against infections of Gram-positive bacteria, which have developed resistance to a number of antibiotic compounds. However, seven of the strains were found to be susceptible to vancomycin and may therefore represent potentially interesting candidates for further probiotics research (Table 6). Nevertheless, further studies must be performed to check antibiotic resistance genes present in plasmids in order to obviate horizontally transferring to microorganisms in the gastrointestinal microbiota and pathogenic bacteria when administered as probiotics.

The colonization effectiveness of isolated strains was evaluated by analyzing hydrophobicity toward xylene and chloroform, which is considered a major determinant of adhesion to epithelial cells of the gut and potential for biofilm formation (67). All 13 isolates exhibited bacterial adhesion to hydrocarbons (BATH) values ranging from 1% to 100%, showing high affinity for xylene and chloroform. Similar studies showed that probiotic *Bacillus* species demonstrate considerable adherence to the gastrointestinal (GI) tract (68). In addition to this, all 13 selected strains showed high binding properties and coaggregation to E. coli ATCC 25922, which may play a role in exclusion of pathogenic microorganisms (69). Moreover, Collado et al. reported that noncoaggregating organisms are easily removed from the GI tract (70). However, other studies reported that these characteristics are strain specific. Thus, selecting probiotics based on higher adhering ability and aggregation ability is not a desirable method (71). This has been also confirmed in our research where a strain with high coaggregation does not necessarily have a high affinity for chloroform and xylene.

In summary, 13 strains out of 250 isolates had the ability to degrade 2 substrates (lactose and gliadin) that cause common digestive disorders. These strains also possessed probiotic features such as resistance to gastric juice and intestinal fluid. These strains and their enzymes may therefore serve as useful candidates for future research in food processing and in reducing the effect of lactose and gliadin digestion in gluten-intolerant patients. However, further purification and characterization of the characteristics of produced proteases are required. Furthermore, studies regarding the presence of antibiotic resistance genes are also needed. This study also demonstrated clearly that novel strains with biotechnological potential can be discovered in pristine subsurface ecosystems.

## MATERIALS AND METHODS

### Sample collection.

Twenty-four different cave sediment samples were collected from 10 different pristine or nearly pristine caves in six different regions in Algeria and at different depths from 0 m to −450 m (Table 1). Samples were collected 10 cm below the surface from the dark zone at each sampling site. We used 50-mL sterile plastic vials to collect samples aseptically (with 5 replicates). During transport, the samples were stored in refrigerated boxes at 4°C. In the laboratory, the samples were stored at 4°C for a maximum of 48 h until they were further processed.

### Cave sediment analyses.

**(i) pH and conductivity.** Sediments were air-dried, crushed, passed through a 2-mm mesh sieve, and mixed with distilled water. After sedimentation, the supernatant was used to determine pH (Hanna Instruments, Sweden) and conductivity (Hanna Instruments). All assays were performed in triplicate.

**(ii) Temperature and humidity.** The temperature was measured with an electronic thermometer and humidity with a hygrometer (OEM HTC-1; Sweden). All assays were performed in triplicate.

**(iii) Organic matter and carbonates.** Total organic matter was measured using the Walkley and Black method (72); total carbonates were measured using the Bernard’s calcimeter method (73). All assays were performed in triplicates.

### Screening of isolates.

**(i) Enrichment and basic screening of isolates.** To ensure the isolation of spores from spore-forming bacteria and to minimize growth of vegetative cells, samples were heat treated at 80°C for 15 min and then chilled on ice, serially diluted up to 10^−2^ times, plated on tryptic soy agar (TSA; Merck) and incubated aerobically for a week at 4, 14, 20, and 25°C to simulate the natural temperature conditions in the different caves. The isolates were further purified by repeated purity streaks. For long-term storage, the isolates were stored in 50% glycerol nutrient broth tubes at −20 or −80°C.

**(ii) Microscopy and sporulation test.** Microscopy (Leica Microsystems, Germany; Zeiss Axioplan 2 imaging) at ×400 to ×1,000 magnification was performed on live cells before and after staining with a 0.1% methylene blue solution. The Gram characteristics were analyzed both by the potassium hydroxide test (74) and the Gram stain (75). To test the sporulation ability of selected strains, a sporulation medium was prepared according to reference 76 with slight modifications: 5 mg/L of MnSO_4_ was added to the culture medium (TSA). Spore formation was evaluated by microscopy.

**(iii) Safety assessment of selected isolates.** Two types of safety assessments were performed on all obtained isolates. To assess hemolytic activity, isolated strains were streaked onto Columbia agar (VWR Chemicals) supplemented with 5% sheep blood and incubated at 37°C for 24 to 48 h. Hemolysis patterns were examined and sorted by observing hydrolysis zones, such as alpha-hemolysis (greenish color), beta-hemolysis (clear zone), and gamma-hemolysis (no reaction) (77). E. coli ATCC 25922 was used as an alpha-hemolysis positive control, Staphylococcus aureus ATCC 43300 as a beta-hemolysis positive control, and Bacillus cereus (Clinical Microbiology, Laboratory Medicine, CHU Constantine, Algeria) as a negative control (gamma-hemolysis). In the lecithinase test, lecithinase activity was analyzed by streaking isolates onto egg yolk agar plates, and they were incubated at 37°C for 24 h for 1 week (78). A positive lecithinase reaction was diagnosed by the appearance of a white, opaque, diffuse zone in the medium surrounding the colonies (77); Bacillus cereus was used as a positive control.

**(iv) Antibiotic susceptibility.** Susceptibility of isolates to antibiotics was carried out by using the disk diffusion method according to the standard method by Bauer (79). Briefly, overnight cultures of approximately 10^9^ CFU/mL^−1^ were swabbed onto Mueller-Hinton agar. Antibiotic-impregnated discs were placed on swabbed plates and incubated at 37°C for 24 h. The inhibition zone diameters were measured, and tests were performed in triplicate. Selection of antibiotics was based on the EFSA guidelines for testing antibiotic susceptibility of *Bacillus* species (40). The tested antimicrobial disks included antibiotics inhibiting protein synthesis (amikacin, 30 μg; ciprofloxacin, 1 μg; erythromycin, 10 μg; and gentamicin, 10 μg), cell wall synthesis (oxacillin, 5 μg; cefazolin, 30 μg; vancomycin, 5 μg; ampicillin, 2 μg; amoxicillin, 10 μg; and imipenem, 10 g), and RNA polymerase action (rifampin, 15 μg) (HiMedia). Results were analyzed based on *Bacillus* breakpoint guidelines of the European Food Safety Authority (40).

### Screening for enzymatic activities.

**(i) Screening for gliadin degrading bacteria.** Strains were streaked onto gliadin plates and prepared according to reference 80. Briefly, gliadin was dissolved in a 60% ethanol solution (molecular grade, 99.8%) and shaken overnight at room temperature. After centrifugation, (2 min at 300 rpm), dissolved gliadin was added to Luria-Bertani agar (Merck). Pseudomonas aeruginosa ATCC 27853 was used as positive control and E. coli ATCC 25922 as negative control. Hydrolysis of gliadin was diagnosed by a clear zone over the colony. The gliadin used in this study was extracted from commercial wheat gluten according to references 81 and 82 as follows. Commercial wheat gluten (WG) powder purchased from Reppe AB (Lidköping, Sweden) was used for the production of gliadins. The WG powder was comprised of 77.7% protein (dry weight; protein content calculated according to NMKL-6 [Kjeltec; nitrogen factor, ×5.7]), 5.8% starch (dry weight, according to Ewart’s method) and 6.9% moisture content (according to total weight; NMKL-23). The WG powder was mixed with 70% ethanol (a blend with Millipore water) with a magnetic stirrer placed on a mechanical shaker (Hunkel Ika; Werk KS 500) for 30 min and 300 rpm at room temperature, and the blend was centrifuged for 10 min at 12,000 × *g* (Beckman centrifuge J2.21) to collect the supernatant (83). The obtained supernatant was further placed in a rotary evaporator under vacuum (Buchi) to remove ethanol. The gliadin powder was collected after centrifugation and freeze-drying (Edwards Modulyo). The protein content of gliadin powder was 91% according to the Dumas method (Thermo Scientific; Flash 2000 NC analyzer) (84).

**(ii) Screening for beta-galactosidase–producing bacteria.** Strains were streaked onto Luria-Bertani (Merck) plates infused with 50 μL X-Gal (5-bromo-4-chloro-3-indole-β-d-galactopyranoside; Thermo Fisher Scientific) and 0.25 mM dimethyl sulfoxide (DMSO; Thermo Fisher Scientific) (GenEon) and incubated at 37°C for 48 h. E. coli ATCC 25922 was used as a positive control and Staphylococcus aureus ATCC 43300 as a negative control. Blue coloration of strains indicates beta-galactosidase production.

**(iii) Beta-galactosidase activity assay.** Selected bacteria were assayed for beta-galactosidase activity by the procedure described in reference 85. Briefly, overnight cultures were harvested by centrifugation (Ortoalresa) at 5,000 rpm for 10 min, washed twice with phosphate-buffered saline (PBS; 10 mM, 130 mM NaCl, pH 7.4), and then resuspended and diluted to 1 mL of Z buffer (50 mL; 0.06 M Na_2_HPO_4_·7H_2_O, 0.04 M NaH_2_PO_4_·H_2_O, 0.01 M KCl, 0.001 M MgSO_4_, and 0.05 M β-mercaptoethanol [BME], pH 7). The optical density of diluted cells was then measured at 600 nm (OD_600_). For cell permeabilization, 100 μL chloroform and 50 μL of 0.1% SDS were added to the culture solution of 1 mL. Ortho-nitrophenyl-*p*-d-galactopyranoside (ONPG; 4 mg/mL = 0.013 M) was used as the substrate for the reaction by adding 0.2 mL to the permeabilized cell and then incubating the mixture at 37°C until a yellow color started developing. The reaction was stopped after sufficient yellow color had developed by adding 0.5 mL of 1 M Na_2_CO_3_. Optical density was then recorded at 420 nm and 550 nm for each tube. The production of a yellow color indicated positive result (85). E. coli ATCC 25922 was used as a positive control and Staphylococcus aureus ATCC 43300 as a negative control. Units of enzyme activity were calculated using the following equation:

$$\text{MU }=\;1,000\;\times \;[({\text{OD}}^{420}-1.75\;\times {\text{OD}}^{550})]/(T\times V\times {\text{OD}}^{600})$$

**(iv) Degradation of gliadin in solution.** Gliadin-hydrolyzing activity of selected bacteria was assayed by using gliadin as a sole source of nitrogen. Two different media were prepared using acid-treated gliadin (to simulate stomach acidity) and nontreated gliadin, respectively. For medium 1, an acid-hydrolyzed gliadin solution was prepared as described in reference 39 by dissolving wheat gliadin at a concentration of 100 mg/mL in 3 mL of 2.5 M HCl (pH 2) and incubating for 1 h at room temperature with occasional shaking. The pH was adjusted to 6.5 with 2 M NaOH, followed by the addition of 60% ethanol (vol/vol) to reach an end concentration of 15 mg/mL gliadin. The solution was incubated for 1 h at 37°C under agitation at 250 rpm. Following this, 20 mL of acid-hydrolyzed gliadin was added to a freshly autoclaved salt solution (39). For medium 2, the gliadin mixture was dissolved to 5 mg/mL in 60% (vol/vol) ethanol and then added to the bacterial solution to produce an end concentration of 250 μg/mL (86). One milliliter of bacterial solution (cell concentration, 10^6^/mL) was inoculated into each medium. Cultures were incubated at 37°C for 48 h, and samples were taken after 0 h, 6 h, 24 h, and 48 h, dried, and analyzed by mini-SDS-PAGE (Bio-Rad Laboratories GmbH, Munich, Germany), according to the protocol given in reference 87, under reducing conditions by using a vertical 13% separating gel and 10% stacking gel polyacrylamide. Staphylococcus aureus ATCC 43300 and Pseudomonas aeruginosa ATCC 27853 served as positive controls, and E. coli ATCC 25922 as a negative control.

**(v) Degradation of gliadin in gel.** Strains were grown in nutrient broth (Difco Laboratories), harvested by centrifugation, and suspended in nonreducing buffer (4% SDS, 20% glycerol, 0.01% bromophenol blue, and 125 mM Tris-HCl). Gliadin zymography was performed as described in reference 88 using a vertical 8% polyacrylamide (under nonreducing conditions). Gels were then processed in a renaturing solution (2.5% Triton X-100) and then incubated in a Triton buffer (1% Triton X-100). After 48 h of incubation at 37°C, gels were stained in 0.2% Coomassie brilliant blue in 40% methanol and distained.

**(vi) Extracellular hydrolase activity.** Extracellular protease production of selected strains was determined by streaking selected bacteria onto agar plates with 1% (wt/vol) skimmed milk. Cultures were incubated at 37°C for 48 h. Colonies surrounded by a clear zone indicated extracellular proteolytic activity (89). Pseudomonas aeruginosa ATCC 27853 was used as positive control.

### Survival in gastrointestinal tract conditions.

**(i) *In vitro* survival in simulated gastric juice.** Vegetative cells were examined for their tolerance to gastric conditions, as described in reference 90, with slight modifications. Briefly, overnight cultures (1% [vol/vol]) were added into a simulated gastric juice (3 g/L of pepsin; 1:3,000; from hog stomach; Biochem; pH 1.5) and incubated at 37°C for 3 h. Cells were serially diluted and enumerated by plate count on nutrient agar (Biotest) plates after 0 and 3 h of incubation at 37°C.

**(ii) *In vitro* survival in simulated intestinal fluid.** To determine the tolerance of selected strains through the simulated intestinal fluid, overnight cultures (1% [vol/vol]) were inoculated into a simulated intestinal fluid (0.5% NaCl solution containing 1 g/L pancreatin [porcine pancreas; Sigma-Aldrich] and 1% bile salts [ox bile; Sigma-Aldrich; pH 8]) and incubated at 37°C for 4 h. Cells were serially diluted and enumerated on nutrient agar plates (Biotest) after 0 and 3 h of incubation at 37°C. CFU per milliliter were calculated and expressed as log_10_ CFU/mL. Tolerance to pH and bile salts and survival rates of cells were calculated as the percentage of CFU after exposure to gastrointestinal conditions compared to the initial CFU (91).

### Cell surface characteristics.

**(i) Auto-aggregation.** Bacterial auto-aggregation ability was assayed according to reference 92 with a slight modification. Fresh bacterial cells were harvested by centrifugation at 6,000 rpm for 10 min, cell pellets were washed twice with PBS (10 mM sodium phosphate buffer, 130 mM sodium chloride, pH 7.4), resuspended in the same buffer, and adjusted to a concentration of 0.5 ± 0.02 at OD_600_. Two milliliters of each cell suspension was vortexed for 10 s and incubated at 37°C for 3 h. After incubation, 1 mL of the supernatant was removed, and the OD_600_ (A3h) was measured. The auto-aggregation percentage is expressed as

$$\text{auto}-\text{aggregation }\left(\text{\%}\right)=1-\left(\frac{\text{A}3\text{h}}{\text{A}0\text{h}}\right)\times 100$$

**(ii) Coaggregation.** Following the protocol of reference 93 with slight modifications, fresh selected strain cells and reference strain (E. coli) were harvested by centrifugation at 6,000 rpm for 10 min, cell pellets were washed twice with PBS (10 mM sodium phosphate buffer, 130 mM sodium chloride, pH 7.4), resuspended in the same buffer, and adjusted to a concentration of 0.5 ± 0.02 at OD_600_. Equal volumes of selected strains and reference strain were mixed and incubated at 37°C for 2 h. The supernatants were measured at 600 nm. The coaggregation percentage was calculated according to the following equation:

coaggregation (%) = [1 − mix A/(probiotic A + pathogen A)/2] ×100

**(iii) Hydrophobicity.** To study cell surface properties, the bacterial adhesion to solvent assay (BATS) was used as described in reference 94 with slight modifications. Two solvents were used, xylene (a polar solvent) to describe hydrophobic surface characteristics and chloroform (polar acidic solvent) to describe electron donor properties. Briefly, overnight cultures were harvested by centrifugation for 15 min at 5,000 rpm, washed twice with neutral PBS, and diluted again in PBS (10 mM sodium phosphate buffer, 130 mM sodium chloride, pH 7.4) to 3 mL. Three milliliters of each cell suspension was mixed with 1 mL of solvent, vortexed, and allowed to settle. After phase separation, the aqueous phase was carefully removed and measured at 600 nm (Shimadzu UV-1280). BATS percentage was expressed as follows:

$$\text{BATS}\;\%=\left(1-\frac{A_{2}}{A_{0}}\right)\times 100$$

### Statistical analysis.

All results are presented as the mean of three different replicates (independent experiments). One-way analysis of variance (ANOVA) was carried out to identify significant differences between means using SPSS (version 12.0; SPSS Inc., Chicago, IL, USA). Significant differences between the means were performed using Duncan’s test with *P* values of <0.05 considered statistically significant.

### Molecular biological methods.

**(i) Fluorescence *in situ* hybridization.** FISH was performed as described in reference 95. Exponentially growing cultures of the strains were fixed with ice-cold absolute ethanol (reaching an end concentration of 50% dissolved in 1× PBS buffer). Prior to FISH, the fixed samples were treated with a modified enzyme treatment compared to reference 41, using two different enzymes, lysozyme (10 mg/mL, 100,000 U/mg; Fluka) and achromopeptidase (2 mg/mL, 100,000 U/mg; Sigma-Aldrich). These two enzymes were added to each bacterial solution and incubated at 37°C for 30 min. For check of purity and confirmation of the phylogenetic position among *Bacillus* within the *Firmicutes*, the following gene probes were used: EUB338 (96), LGC354A, LGC354B, and LGC354C at a formamide concentration of 35%. Samples were viewed using a Leica fluorescence microscope (Germany; using the image software Zeiss Axioplan 2 Imaging). Two kinds of controls were employed, employing FISH without gene probes to explore the natural background autofluorescence and employing the gene probe LGC354C, which should not target taxa targeted by the LGC354B probe.

**(ii) DNA extraction.** Genomic DNA was extracted from pure cultures harvested in the exponential stage, using a modified version of the Griffith DNA extraction protocol (97). The extraction buffer was mixed with 30 μL of 2 mg/mL achromopeptidase (100,000 U/mg; Sigma-Aldrich) and 10 μL of 10 mg/mL lysozyme (100,000 U/mg; Fluka) and incubated for 30 min at 37°C, followed by two cycles (15 s, 4.0 m/s) of bead beating (Nordic Biolabs). After adding 400 μL isoamyl alcohol–phenol-chloroform to this solution, it was vortexed and centrifuged at 13,000 rpm for 15 min. The supernatant was transferred to a new Eppendorf tube, and 400 μL of chloroform-isoamyl alcohol (CHISAM) was added. This solution was vortexed and centrifuged at 15,000 rpm for 5 min. The supernatant was transferred to a new Eppendorf tube, and 3 m sodium acetate (at the ratio of 90 μL/mL supernatant) and ice-cold isopropanol (at the ratio of 200 μL/mL solution) were added. This solution was incubated overnight at room temperature. After this, the solution was centrifuged at 13,000 rpm for 10 min at 4°C. The supernatant was removed, and the pellet was washed with 70% ethanol (molecular grade) and centrifuged at 13,000 rpm for 2 min. The supernatant was removed, and the pellet was let dry. Finally, the extracted DNA was rehydrated in 30 μL sterile Milli-Q water and incubated overnight at 4°C. The DNA concentration was measured using a NanoDrop (Thermo Fisher Scientific). The quality of the DNA was evaluated by gel electrophoresis in combination with the 1 kb Plus DNA ladder (Thermo Fisher Scientific). DNA samples were stored at −20°C.

**(iii) PCR and gene sequencing.** 16S rRNA gene fragments were amplified using the general primer pair 8F-1492R (8F, 5′-AGAGTTTGATCCTGGCTCAG-3′; 1492, 5′-GGTTACCT TGTTACGACTT-3′) (98). The Phusion high-fidelity Taq polymerase (Thermo Fisher Scientific) was used for *in vitro* amplification of 16S rRNA gene fragments. An initial denaturation step at 98°C for 1 min was followed by 35 cycles of denaturation for 10 s at 98°C and chain extension for 1 min at 72°C. The annealing temperature was 15 s at 55°C for the 16S rRNA primer pair. PCR products were purified with the purification kit (QIAquick; Qiagen) following the manufacturer’s instructions. The PCR was evaluated by gel electrophoresis, using the BioRAD and Quantity One Bio-Rad software and GeneRuler (1 kb Plus; Thermo Fisher Scientific). Sanger sequencing of the PCR amplificates was performed at Eurofins Genomics (https://eurofinsgenomics.eu/).

**(iv) Phylogenetic evaluation.** The 16S rRNA gene sequences (minimum length > 1,000 nucleotides) retrieved in this study were evaluated by the bioinformatics software package ARB (99), using two kinds of reference gene sequence data, (i) the Living Tree Project 16S rRNA gene database (version 132), containing 13,914 high-quality gene sequences only from culturable species (https://www.arb-silva.de/no_cache/download/archive/previous_living_tree/); and (ii) the SILVA database version 138.1 (100, 101), containing 304,571 gene sequences from culturable, as well as nonculturable, organisms, affiliated with selected taxa within *Bacillales*, including some outgroups within *Firmicutes*. Both trees contained large orientation trees based on the parsimony treeing method. New gene sequences were roughly aligned in the SILVA online alignment tool (https://www.arb-silva.de/aligner/) and then visually further refined with the ARB software. A general phylogeny of the 13 gene sequences retrieved in this study was reconstructed using three treeing methods (neighbor-joining distance method, maximum parsimony, and randomized a[x]ccelerated maximum-likelihood methods) (102), which were used to reconstruct a consensus tree. A large orientation tree (based on the two types of databases created) was used to identify the closest culturable as well as nonculturable relatives.

### Data availability.

Thirteen of the strains have been deposited to the German Collection of Microorganisms and Cell Cultures GmbH (DSMZ; https://www.dsmz.de/) under the accession numbers as follows: F9, DSM 113758; F10, DSM 113759; J4, DSM 113760; T1, DSM 113761; Dj16, DSM 113762; Dj17, DSM 113763; D3, DSM 113764; BS16, DSM 113765; A9, DSM 113766; A9, DSM 113767; SSI5, DSM 113768; BS13, DSM 113769; and BS14, DSM 113770. Partial sequence data were submitted to GenBank under the following accession numbers: A8, OL823170; A9, OL823171; BS16, OL823172; D3, OL823173; Dj16, OL823174; Dj17, OL823175; F9, OL823176; F10, OL823177; J4, OL823178; SSI5, OL823179; T1, OL823180; B13, OM146734; and B14, OM250505.
